# Dynamic interplay between corticosteroid treatment and the role of SRC-1 gene dysregulation in the progression of WHO-Grade 4 Astrocytoma

**DOI:** 10.1007/s11060-023-04385-5

**Published:** 2023-07-04

**Authors:** Maher Kurdi, Motaz M Fadul, Bassam M. J. Addas, Eyad Faizo, Shadi Alkhayyat, Ahmed K. Bamaga, Taghreed Alsinani, Yousef Katib, Fahad Okal, Yazid Maghrabi, Abdulrahman J. Sabbagh, Rana Moshref, Sultan Albalawi, Alaa Alkhotani, Taher F. Halawa, Nasser Mulla, Sahar Hakamy, Saleh Baeesa

**Affiliations:** 1 Department of Pathology, Faculty of Medicine, King Abdulaziz University, Rabigh, Kingdom of Saudi Arabia; 2 Neuromuscular Unit, King Fahad Medical Research Center, Jeddah, Saudi Arabia; 3 Department of Surgery, Faculty of Medicine, King Abdulaziz University, Jeddah, Saudi Arabia; 4 Department of Surgery, Faculty of Medicine, University of Tabuk, Tabuk, Saudi Arabia; 5 Department of Internal Medicine, Faculty of Medicine, King Abdulaziz University and Hospital, Jeddah, Saudi Arabia; 6 Department of Paediatric, Faculty of Medicine, King Abdulaziz University, Jeddah, Saudi Arabia; 7 Department of Neurosurgery, King Fahad General Hospital, Jeddah, Saudi Arabia; 8 Department of Radiology, Faculty of Medicine, Taibah University, Madinah, Saudi Arabia; 9 Department of Neuroscience, Neurosurgery Section, King Abdulaziz Medical City, National Guard Health Affairs, Jeddah, Saudi Arabia; 10 Department of Neuroscience, King Faisal Specialist Hospital and Research Center, Jeddah, Saudi Arabia; 11 Department of Pathology, Faculty of Medicine, Umm Al-Qura University, Makkah, Saudi Arabia; 12 Department of Paediatric, Faculty of Medicine, King Abdulaziz University, Rabigh, Saudi Arabia; 13 Department of Internal Medicine, Faculty of Medicine, Taibah University, Medina, Saudi Arabia

**Keywords:** WHO Grade 4 Astrocytoma, Corticosteroid, CD8, T-cells, SRC-1

## Abstract

**Background:**

Corticosteroid is commonly used before surgery to control cerebral oedema in brain tumours and is frequently continued throughout treatment. Its long-term effect of on the recurrence of WHO-Grade 4 astrocytoma remains controversial. The interaction between corticosteroid, SRC-1 gene and cytotoxic T-cells has never been investigated.

**Methods:**

A retrospective cohort of 36 patients with WHO-Grade 4 astrocytoma were examined for CD8 + T-cell and SRC-1 gene expressions through IHC and qRT-PCR. The impact of corticosteroid on CD8^+^T-cells infiltration, SRC-1 expression, and tumour recurrence was analyzed.

**Results:**

The mean patients age was 47-years, with a male to female ratio 1.2. About 78% [n = 28] of the cases showed reduced or no CD8^+^T-cell expression while 22% [n = 8] of cases have showed medium to high CD8^+^T-cell expression. SRC-1 gene was upregulated in 5 cases [14%] and 31 cases [86%] showed SRC-1 downregulation. The average of total days and doses of administered corticosteroid from the preoperative period to the postoperative period was at range of 14–106 days and 41–5028 mg, respectively. There was no significant statistical difference in RFI among tumours expressing high or low CD8^+^T-cells when corticosteroid was administered in recommended or exceeded doses [p-value = 0.640]. There was a significant statistical difference in RFI between CD8^+^T-Cell expression and SRC-1 gene dysregulation [p-value = 002]. Tumours with high CD8^+^T T-cell expression and SRC-1 gene downregulation had late recurrence.

**Conclusions:**

Corticosteroid treatment can directly affect the SRC-1 gene regulation but does not directly influence cytotoxic T-cells infiltration or tumor progression. However, SRC-1 gene downregulation can facilitate late tumor recurrence.

## Introduction

The 5th edition of World Health Organization [WHO] of Central Nervous System [CNS] and European Association of Neuro-oncology [EANO] has subclassified WHO-Grade 4 astrocytoma into IDH-mutant and IDH-wildtype categories. Specifically, IDH-wildtype astrocytoma has been identified as a glioblastoma [1, 2]. Treating patients with WHO-Grade 4 astrocytoma is a challenging task, given the median survival rate of the disease of about 14–15 months even with surgical resection and combined radiotherapy and chemotherapy, attending sometimes the survival of 18 months and rarely up to two years [3–6]. The progression of the disease is influenced by several factors such as patient age, extent of resection, co-morbidities, and IDH mutational status, all of which have been demonstrated to affect the outcome [1]. One of the recently investigated factors is the administration of corticosteroid in glioma patients during adjuvant therapies. Because steroid particles may reach tumour microenvironment, the impact of these molecules on tumour infiltrating lymphocytes, mainly Cytotoxic T-cells, and other gene receptors has not been fully explored.

Dexamethasone [DEX] is a potent synthetic corticosteroid that is commonly used to treat vasogenic edema in patients with brain tumours. However, its effect on survival has not been investigated in clinical randomized trials [7, 8]. In 1952, The use of corticosteroids to treat cerebral edema in patients with brain tumours was first initiated by Ingraham. In 1957, Kofman utilized prednisone to treat peritumoral edema in brain metastases [9]. Preoperative corticosteroid treatment usually relief symptoms of cerebral edema within 48 h [10]. It is commonly administered after surgery and prior to initiating radiotherapy in individuals whose tumors exhibit a substantial mass effect. DEX has a minimal mineralocorticoid activity, long half-life, and high potency. Despite its common use in clinical practice, few guidelines have set out the requirements and the optimal dose of corticosteroid treatment in patients with brain tumour. For patients experiencing symptoms, it is recommended to administer a maximum daily dose of 16 mg corticosteroid, divided into four equal doses, following surgery [11–13]. Two reviews have recommended an initial loading dose of 10–20 mg DEX when a patient presents with acute symptoms caused by a brain tumour followed by maintenance dosing with DEX in divided doses up to 16 mg daily [14, 15]. Patients should be carefully monitored for endocrine, muscle, gastrointestinal, psychiatric, and hematologic complications. As the glucocorticoid receptor binding site is present in the BAFF (B-cell activating factor) promoter region, but not in the APRIL promoter region, it is advised to detect BAFF levels at an early stage. Nonetheless, administering a combination of corticosteroids and vitamin D3 could potentially reduce APRIL serum levels [16]. DEX should therefore be tapered down once symptoms begin to improve and before radiotherapy starts. Some studies have recommended tapering when a dose equivalent to 16–20 mg of DEX daily is attained [17]. Patients who have high-grade glioma, are symptomatic, or have poor life expectancy can also be continued on DEX [0.5–1.0 mg] daily after radiotherapy. It is also recommended that DEX being discontinued before starting adjuvant chemotherapy however, this decision is determined by the treating physician [18].

The impact of corticosteroids on cellular growth in glioma models and patient survival remains a controversial topic. The experimental evidence is limited and insufficient to draw conclusive results. Shield et al. reviewed 73 glioblastoma patients who received radiotherapy and chemotherapy with DEX, and they found that using DEX during adjuvant therapies have been correlated with poor overall survival [OS] [19]. Furthermore, there are scattered reports suggesting that DEX dependency during radiotherapy can be an independent poor predictor of survival in glioblastoma [20, 21]. According to Watnie et al.‘s findings, patients who required corticosteroids after surgery had a 1.9 times greater risk of death compared to patients who did not require steroids following their operation [21]. It was found that corticosteroid use during radiotherapy is an independent indicator of shorter OS [9, 22]. Studies also revealed that patients who did not receive DEX at the beginning of radiotherapy had a median survival of 20 months, while patients who were on DEX had a survival time of 13 months [9, 22].

The exact mechanism by which steroids reduce peritumoral edema is not fully understood. However, it is believed that DEX possesses anti-inflammatory properties that can inhibit T-cells in the microenvironment. This inhibition may prevent the evolution of tumour-associated macrophages [TAMs] into tumour cells [23]. It is also not clear whether DEX impacts the effectiveness of DNA-damaging therapy for WHO-Grade 4 astrocytoma [24]. Studies examining the relationship between T-cell infiltration and prognosis in glioblastoma have yielded conflicting results. While some studies indicated that there was a correlation between T-cells and the survival of glioblastoma patients, others did not support this association. The accumulation of CD8 cytotoxic T-cells in the glioma microenvironment may lead to faster eradication of tumour cells [25–28]. Mouldine et al. considered CD8 cytotoxic T-cells as independent predictor of OS in patients with glioblastoma. This mechanism may not work all the time considering the relationship between TILs and TAMs. Kurdi at al found that when TAMs enclose tumour cells, they prevent cytotoxic T-cell to identify and supress tumour cells [29]. This may cause tumour cells evolution. The regulation of CD8 cytotoxic T-cells infiltrating the microenvironment has not been previously explained. However, other unknown factors may downregulate cytotoxic T-cells. The association between T-cell regulation and corticosteroid treatment in patients with WHO-Grade 4 astrocytoma has not yet been investigated, although the cellular immune response in relation with glucocorticoids, often suppressive action of glucocorticoids, was signaled in literature [30, 31]. It is still unclear whether the injected DEX targets a specific gene receptor in the glioma microenvironment, and it is also uncertain if the cells in the microenvironment are affected uniformly.

**Fig. 1:**
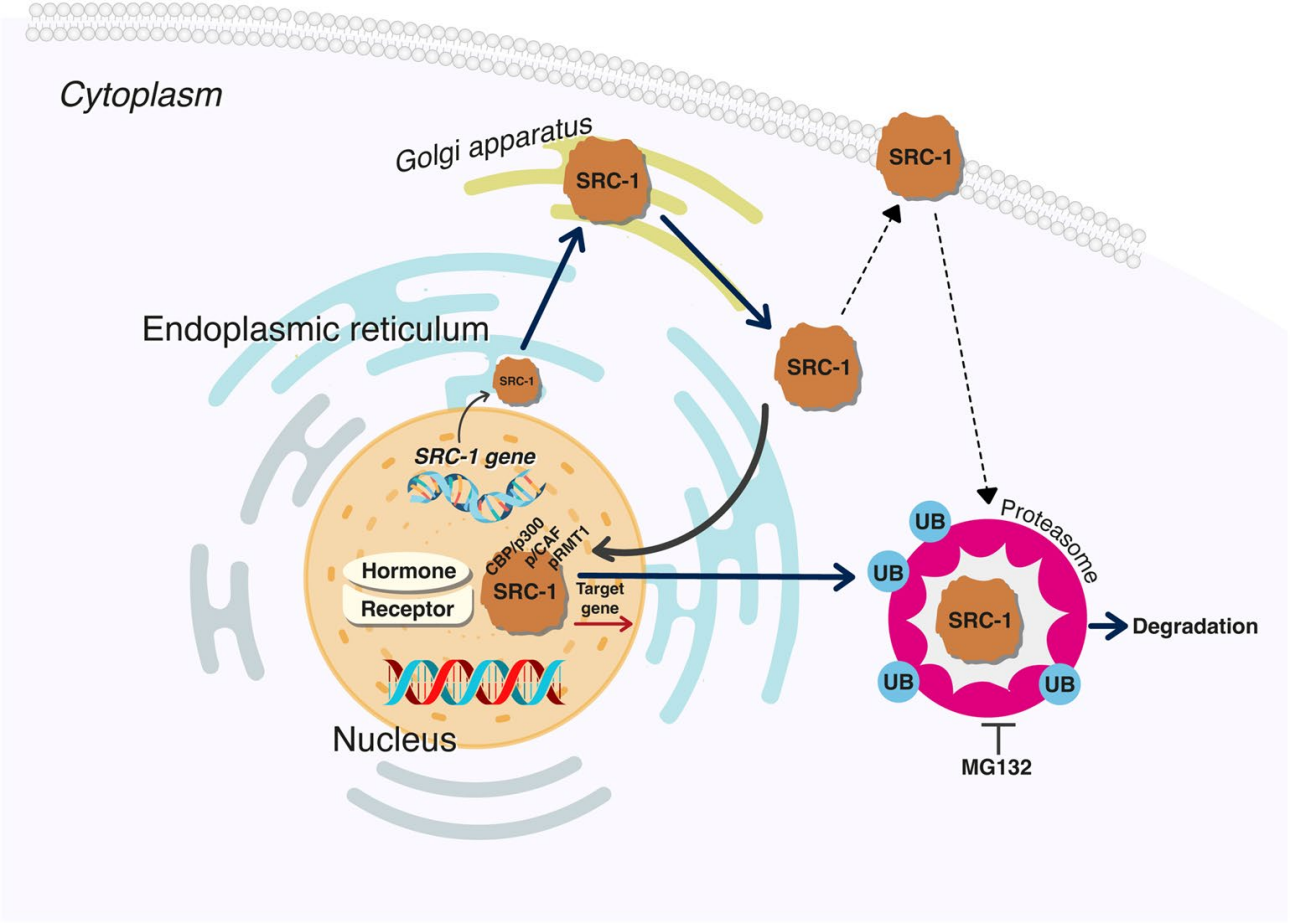
Schematic illustration of the transcriptional regulation and synthesis of SRC-1 gene. After transcription, mRNA is matured through rough endoplasmic reticulum and translocated in the cell nuclei to co-activate the targeted receptor in the nucleus. To regulate the gene transcription, SRC-1 transcriptional complex is formed to contains the cAMP response element binding protein [CREB]-binding protein [CBP], p300, and the p300/CBP-associated factor [p/CAF] as well as protein arginin methyltransferases 1 [PRMT1]. To stop its function, SRC-1 translocates to the proteasomes and gets degraded by ubiquitination. The degradation of SRC-1 can be terminated by MG132.

One of the common genes associated with steroid interaction is steroid receptor coactivator [SRC]. SRC is a one of p160 steroid receptor gene family that contains three major members, namely SRC-1, SRC-2, and SRC-3 [32]. The identification of SRC-1 was initially based on its ability to increase transcriptional activity of receptors located at nuclear membrane. SRC-2 and SRC-3 were subsequently discovered due to their frequent amplification in breast and ovarian cancers and their homology to SRC-1. The SRC-1 protein has a size of approximately 160 kDa and regulates the ligand-dependent transactivation of several nuclear receptors such as estrogen, androgen, and thyroid receptors [33, 34] (Fig. 1). Its intracellular ubiquitination regulates the termination of hormone action. SRC-1 has been explored in the brain tissue, and mainly located in the ventromedial nucleus of the thalamus and hippocampus [35, 36]. Its normal physiological role in brain development, memory and learning has been reported [37]. SRC-1 has been found in high-levels in the brain compared to SRC-2 and SRC-3 [35, 38]. In the adult mouse brain, SRC-1 mRNA was upregulated in many subcortical structures in the brain [39].

Additionally, the detection of SRC-1 expression in astrocytic tumors provides further evidence for the presence of SRC-1 in glial cells [40]. Its expression in astrocytomas indicates the potential role of SRC-1 in progression of astrocytomas. Nevertheless, SRC-2 and SRC-3 were also discovered to be highly expressed in astrocytic tumors [41]. The association between corticosteroid treatment given for patients with brain tumour and SRC-1 has not ever been discussed in the literature.

Our current study investigated the impact of corticosteroid injections on the function of cytotoxic T-cells and the regulation of the SRC-1 gene in the microenvironment of WHO-Grade 4 astrocytoma, as well as the impact of this relationship on tumor recurrence.

## Materials and methods

### Patients selection

The use of patient samples in this research study has been authorized by the Biomedical Ethics Committee at King Faisal Specialist Hospital and Research Center [CA-2020-06] and King Abdulaziz University [HA-02-J-008]. Our study included 36 patients diagnosed histologically with WHO-Grade 4 astrocytoma after complete surgical resection, in the period between 2016 and 2018 (Table 1). The histological diagnosis has been revisited by a certified neuropathologist [MK/AK], based on 2021-WHO classification of CNS tumours [1, 29]. All tumours showed tissue necrosis and microvascular proliferation.

**Table 1:** Biological data of the 36 tumour cases enrolled in our study. IDH: Isocitrate dehydrogenase; SRC-1: Steroid Receptor coactivator-1; TDOS: Total dose of administered steroid; TDS: Total days of administered steroid; RFI: Recurrence free-interval

Age	Sex	IDH Status	CD8 T-cell	SRC-1 Expression	TDOS	TDS	Chemotherapy	RFI
14	F	IDH-mutant	No expression	Upregulated	3260.5	381	TMZ +	149
60	F	IDH-mutant	No expression	Upregulated	1187	158	TMZ	163
52	F	IDH-wildtype	Low Eepression	Upregulated	2631	171	None	177
31	M	IDH-mutant	No expression	Upregulated	441	105	TMZ	361
21	M	IDH-mutant	Medium expression	Upregulated	424	53	TMZ	322
31	M	IDH-mutant	Low expression	Downregulated	3812.1	745	TMZ +	684
34	F	IDH-mutant	Medium expression	Downregulated	5028	508	TMZ	651
58	M	IDH-mutant	No expression	Downregulated	3389	382	TMZ	213
66	F	IDH-mutant	Medium expression	Downregulated	1524.2	1065	TMZ	623
43	M	IDH-wildtype	Low expression	Downregulated	2042	250	TMZ	475
44	F	IDH-wildtype	Medium expression	Downregulated	1763.5	235	TMZ	302
69	F	IDH-wildtype	Medium expression	Downregulated	1211	238	TMZ	534
25	M	IDH-mutant	High expression	Downregulated	1890	233	TMZ	639
85	M	IDH-wildtype	High expression	Downregulated	1124.6	229	TMZ	850
47	M	IDH-wildtype	Medium expression	Downregulated	800	203	TMZ	747
55	M	IDH-mutant	Low expression	Downregulated	672	192	TMZ +	481
61	F	IDH-mutant	Low expression	Downregulated	848	170	TMZ	552
46	M	IDH-wildtype	Low expression	Downregulated	1140.8	167	TMZ	171
29	M	IDH-mutant	Low expression	Downregulated	1352.9	163	TMZ	229
64	M	IDH-wildtype	Low expression	Downregulated	1118	130	TMZ	372
45	M	IDH-mutant	Low expression	Downregulated	911	106	TMZ +	176
28	M	IDH-mutant	Low expression	Downregulated	1063.5	99	TMZ	357
27	F	IDH-mutant	Low expression	Downregulated	565.2	96	TMZ	372
59	F	IDH-mutant	Low expression	Downregulated	753	91	TMZ +	534
29	F	IDH-mutant	Low expression	Downregulated	762	60	TMZ	548
54	M	IDH-wildtype	Low expression	Downregulated	178	51	TMZ	33
56	F	IDH-wildtype	Low expression	Downregulated	1158	148	TMZ	485
35	F	IDH-wildtype	Low expression	Downregulated	212.3	59	TMZ	723
83	M	IDH-wildtype	Low Expression	Downregulated	410.4	38	None	217
51	M	IDH-wildtype	Low Expression	Downregulated	266.8	59	None	135
55	M	IDH-wildtype	Low Expression	Downregulated	215.6	43	None	604
64	F	IDH-wildtype	Low Expression	Downregulated	246.4	41	TMZ	191
60	F	IDH-wildtype	No Expression	Downregulated	41	34	TMZ	186
38	M	IDH-mutant	Low Expression	Downregulated	295.5	37	TMZ	261
57	Male	IDH-wildtype	Low Expression	Downregulated	204	14	TMZ +	311
41	Male	IDH-wildtype	Low Expression	Downregulated	445	36	TMZ	270

*IDH* Isocitrate dehydrogenase, *SRC*-1 Steroid Receptor coactivator-1, *TDOS* Total dose of administered steroid, *TDS* Total days of administered steroid, *RFI* Recurrence free-interval

Patients’ data were obtained from hospital records and included patient’s age at diagnosis, gender, location of tumour, IDH1^R132H^ mutation status, treatment plan, and recurrence-free interval [RFI] (Table 1). The RFI was estimated from the beginning of the complete surgical resection to the first day of tumour recurrence.

Patients presented with peritumoral vasogenic edema were treated with corticosteroid, dexamethasone [DEX], prior to surgery at rate between 3 and 16 mg daily. All patients were treated with radiotherapy, and some of them were selected to have adjuvant chemotherapy. A total dose of 60 Gy was administered during radiotherapy, and the post-surgical chemotherapy regimen adhered to the Stupp protocol [5]. Temozolomide [TMZ] was given at rate of 150–200 mg/m^2^ for five days for 6–12 cycles. The patients were clustered into two groups, based on the total dose and days of administered steroid. The total dose of administered steroid was calculated as “preoperative dose + postoperative dose” along therapy course. The usual rate of administered DEX before surgery was 16 mg per day and for 3–5 days. DEX has been tapered down when symptoms began to improve and before radiotherapy started. Symptomatic patients after radiotherapy or who had a poor life expectancy have been maintained on a 0.5–1.0 mg dose of DEX daily after diagnosis, which was totally considered for 4–6 weeks. Some symptomatic patients continued using steroid along the chemotherapy cycle, up to two years. In our study, we clustered the patients into two groups based on recommended dose of the administered steroid that should be given to the patients before or after the surgery. The cutoff value for the total dose and days of steroid was calculated as the following:

- *For the doses of steroid [Preop: 50 mg; Postop: 600 mg; Total [pre + post]: 800 mg.*
- Preop: Group 1 [≤ 50] 22 patients (61.1%); Group 2 [> 50] 14 patients (38.9%).
- Postop: Group 1 [≤ 600] 14 patients (38.9%); Group 2 [> 600] 22 patients (61.1%).
- Total: Group 1 [≤ 800] 17 patients (74.92%); Group 2 [> 800] 19 patients (52.8%).
- *For the total days of steroid cutoff [Preop: 5 days; Postop: 50 days, Total: 60 days].*

### Tissue processing

A 4 μm thick section and tissue rolls were cut from formalin-fixed paraffin-embedded [FFPE] blocks of 36 tumours. The thick section slides were used to process the immunohistochemistry [IHC] for anti-CD8 antibody, and the tissue rolls were utilized for RNA extraction to evaluate SRC-1 gene expression by using Real Time-Polymerase Chain Reaction [RT-PCR].

### Immunohistochemistry [IHC] for anti-CD8 antibody

Anti-CD8 antibody [Rabbit monoclonal, SP16 Clone, ThermoFisher Scientific Invitrogen, Cat# MA514548] directed against human antibody has been used in IHC for 36 unstained slides. The Ultra-View Detection Kit from Ventana was used for the assay, and it was processed in the GX Ventana automated stainer [Tuscon, AZ, USA]. The protocol involved deparaffinization with EZ Prep at 75 °C, heat pre-treatment in a cellular medium for 60 min, followed by incubation at an optimal temperature of 37 °C for 20 min. The antibody was dilute to 1:100. After the mentioned assay, the slides underwent counterstaining with Hematoxylin II and bluing reagent for 30 min, and a section of normal tonsillar tissue was used as a positive control.

The Anti-CD8 antibody is used to stain the tumor-infiltrating lymphocytes [TILs], specifically the CD8 cytotoxic T-cell, in the microenvironment of astrocytoma. Using light microscopy, each section was initially screened at low magnification [x10], and subsequently, two focal non-necrotic areas with anti-CD8 expression were selected for further examination at a higher magnification field [x25]. Cells expressing anti-CD8 was considered as CD8 T-cells positive while the total cells was defined as cells with both expressed CD8^+^T-cell and non-stained cells. In each selected area, the labelling index [LI] of CD8^+^T-cell expression was assessed through the following equation:

$${\text{Labelling Index }}[\% ]{\text{ }} = {\text{ }}\left[ {\frac{{\left( {{\text{CD}}8^{ + } {\text{ T}} - {\text{cells}}} \right)}}{{\left( {{\text{Total cells}}} \right)}} \times 100} \right].$$

**Fig. 2:**
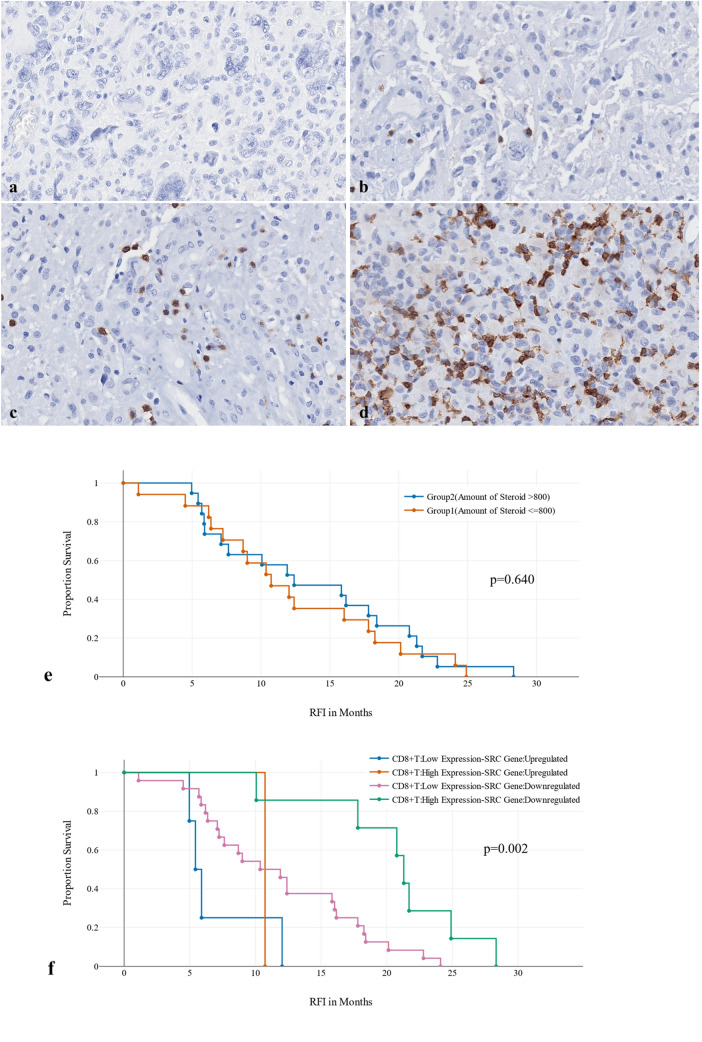
**a**–**f** Anti-CD8 stains cytotoxic T-cells in tumour microenvironment using IHC Magnification [x25]. Four staining patterns were defined: **a** no expression [0%] **b** low expression [< 30%], **c** medium expression [30–50%], **d** high expression [> 50%]. For the relationship between CD8, SRC-I and RFI, **e** There was no significant statistical difference in RFI among the two group of patients [Group 1: <800 mg, Group 2: >800 mg] administered with DEX up to 1-year after surgery; **f** There was a significant statistical difference between CD8^+^T-Cell expression and SRC-1 gene in tumour microenvironment

The average count of two examined areas was taken by the following equation: [Area (1) %Area (2) %] divided by 2. This protocol is typical to the protocol used by Mauldin et al. [25]. Four staining patterns were defined: no expression [0%], low expression [< 30%], medium expression [30–50%], and high expression [> 50%] (Fig. 2a–d)

### RNA extraction

RNA was extracted from 36 tumour samples and two controls. The extraction used the RNeasy FFPE Kit [QIAGEN 73,504] according to manufacturer’s protocol. All centrifugation steps were performed at 8000 g for 15 s at room temperature, unless otherwise stated. Briefly, each FFPE sample was transferred to an Eppendorf, RNase-free, 1.5mL tube and deparaffinized after vertexing for 30 s with 1mL of 99% m-Xylene [Sigma-Aldrich 18,556]. Samples were centrifuged at 17,000 g for 2 min to pellet the samples and the supernatant was discarded. The pellet was washed with 1mL ethanol [100%] and centrifuged at 17,000 g for 2 min; to ensure the samples are completely clean of m-Xylene, the ethanol-washing step was repeated twice. The ethanol was dried off the samples in a 37 °C dry bath for 10 min with the lid open. 150µL Buffer PKD was mixed thoroughly with the pellet and followed with 10µL Proteinase K. Samples were incubated at 56 °C for 15 min; followed by incubation at 80 °C for 15 min and 3 min on ice. The tubes were centrifuged at 18,000 g for 15 min to pellet insoluble tissue debris and the supernatant was transferred to a clean, RNase-free 1.5mL tube. The supernatant was mixed with 16µL DNase Buffer followed by 10µL DNase; the DNase was mixed gently by pipetting and left to incubate for 15 min at room temperature. 320µL Buffer RBC was vortexed with the sample and 720µL 500µL ethanol [100%] was added and mixed by pipetting. 700µL of the lysate-ethanol mixture was transferred to a spin column and centrifuged. The flow-through in the collection tube was discarded in an appropriate waste container and the spin column was replaced in the collection tube. The remaining lysate-ethanol mixture was added to the spin column, centrifuged, and the flow-through was discarded. 500µL of Buffer RPE was added to the spin column, centrifuged, and flow-through was discarded. Washing with Buffer RPE was repeated, then the spin column was placed in a new collection tube and centrifuged empty with the lid open at 17,000 for 5 min. The dry spin column was transferred to an RNase-free 1.5mL tube. 20µL of RNase-Free Water was added centrifuged for 1 min at 17,000 g. 1µL of the sample was used from the RNA-containing eluates for spectrophotometric analysis. The rest of the eluate [~ 19µL] was stored immediately at − 20°.

### Complementary DNA [cDNA] synthesis

cDNA was synthesized using the High-Capacity cDNA Reverse Transcription Kit [Applied Biosystems™, 4,368,814] according to manufacturer’s protocol. Briefly, a master mix was prepared with 1µL RT Buffer, 0.4µL dNTP Mix [100mM], 1µL RT Random Primers, and 1µL MultiScribe™ Reverse Transcriptase was mixed with 70ng of RNA, and the final volume was adjusted to 10µL with RNase-free water. For samples whose concentration was < 10ng/µL, the maximum volume of RNA was added [7.1µL]. After cDNA synthesis, 170µL RNase-free water was added.

### Gene sequencing using RT-PCR

The PCR primers for the experimental targeting gene [SRC-1] and one reference gene, Glyceraldehyde-3-Phosphate Dehydrogenase [GAPDH], were pre-designed [PCR-CDA-HAS-SRC-11]. The following primer sequence for SRC-1 was used: *Forward* 5′-GAGACCACGAAAGGTGCCTAC-3′ and *Revers* 5′- CCCTTGGCGTTGTCGAAGTC − 3 (Amplicon 56).

Real-time PCR was performed using the EverGreen Universal qPCR Master Mix [Haven Scientific, PCR5505], according to manufacturer’s protocol in triplicate reactions. Briefly, 4.9µL of the resulting cDNA was mixed with 5µL of the EverGreen master mix and the appropriate volume of each oligo for a final PCR reaction volume of 10µL, and final reaction volumes was in the 0.2mL qPCR 96-Well Plate, Semi-Skirted plates from Haven Scientific [PCR-SSP-02]. Plates were sealed with the Real-Time PCR Optical Adhesive Seal from Haven Scientific [PCR-OS-0011]. The plates were run on the QuantStudio3 system using the following protocol: 3 min at 95 °C, followed by 40 cycles of 95 °C for 15 s then 60 °C for 60 s [data collection]. To eliminate inter-run variabilities, all assays for a given sample were run on the same plate.

For the analysis, three replicates of threshold cycle [C_T_] values were considered for both targeting gene and reference gene. The mean C_T_ and standard deviation for the reference [GAPDH] and target [SRC-1] genes were calculated from the RT-PCR data and analyzed by ∆∆C_T_ and ∆C_T_ methods. Using the Step One System and Data Assist software, the average C_T_ for both the control and tested genes were calculated from the generated RT-PCR data. Subsequently, the C_T_ of the target gene was normalized to the C_T_ of the reference gene. The ∆CT of the test sample was then normalized to the ∆C_T_ of the control sample, and based on this information, the relative quantification [Rq] and differential expression [fold change, FC] were calculated [1]. ΔC_T_ for Ctrl or test = C_T_ target gene – C_T_ reference gene, [2] ΔΔC_T_ = ΔC_T_ test sample – ΔC_T_ control sample, [3] Relative quantification [Rq] = 2 ^−∆∆CT^ = value. The fold change [differential expression] was also calculated. ΔC_T_ values for each sample were determined using: ΔC_T_ = [mean C_T_ reference gene – mean C_T_ target gene. The results of gene expression are illustrated in (Table 1).

### Statistical analysis

Our statistical analysis was performed to explore the effect of corticosteroid administrated to the patients and its effect on CD8 cytotoxic T-cell and SRC-1 gene regulation, the Fisher’s exact test has been used. A two-tailed t-test for independent samples [equal variances assumed] was applied to compare the RFI by the amount of administrated corticosteroid [preoperative, postoperative and the total amount administered in the entire period]. Kaplan-Meier curve [KMC] and log-rank test were used to compare the distribution of RFI with SRC-1 expression, CD8 cytotoxic T-cells and the total amount of administered corticosteroid in the entire period. A p-value of < 0.05 was considered statistically significant. All statistical analyses in this study were performed using IBM SPSS ver. 24 [SPSS Inc., Chicago, IL, USA].

## Results

The average age of participants was 47 years, with a male to female ratio of 1.2. Most of the tumours [69%, n = 25] were located in the frontal-parietal area and the rest [31%, n = 11] were located in occipital, temporal and cerebellar area. The total dose and the total days of administered corticosteroid for all patients were calculated. IDH1^R132H^ were equally distributed among all cases. CD8^+^T-cell expression was absent in 5 cases [13.9%], low in 23 cases [63.0%], medium in 6 cases [16.7%] and high in 2 cases [5.6%] (Table 1). All Cases with low and no CD8 expression [n = 28] were merged as “low CD8^+^T-cell expression”. All cases with medium and high CD8 expression [n = 8] were merged as “high CD8^+^T-cell expression”. SRC-1 gene was upregulated in 5 cases [14%] and 31 cases [86%] showed SRC-1 gene downregulation. The mean of the total days of administered steroid from the preoperative period to the postoperative period was at range of 14–106 days. The mean of the total dose of administered steroid from the preoperative period to the postoperative period was at range of 41-5028 mg. Isolated radiotherapy was given only for 4 [11%] cases and 32 [89%] cases had combined radiotherapy and chemotherapy. TMZ was given to 26 [72.2%] cases and 10 [27.8%] cases received TMZ with other chemotherapeutic agents. The mean RFI was 391.6 days.

### The relationship between CD8^+^T-Cells expression and RFI among different groups

 There was no significantly statistical difference in RFI [p-value = 0.640] among tumours expressing high or low CD8^+^T-cells when corticosteroid [DEX] has been administered in recommended or exceeded doses before or after the surgery (Table 2). Cases [n = 17] who received exceeded doses of DEX and their CD8^+^T-cells were highly expressed had a RFI between 8 months and 2 years. Moreover, there was no significantly statistical difference in RFI among the two group of patients [Group 1: < 800 mg, Group 2: > 800 mg] administered with DEX from preoperative period and up to 1-year after surgery [p-value = 0.640] (Fig. 2e).

**Table 2:** The relationship between CD8+T-cells Expression and RFI among different groups of patients taking corticosteroid [DEX]. Patients who have received corticosteroids continuously for up to 1- year showed delayed RFI in tumors with low CD8+T-Cell expression and downregulated SRC-1 gene

		Pre-OP Amount steroid groups				
			Group1[ ≤ 50 mg]	Group2[> 50 mg]	Total	p-value
CD8^+^T-cell expression		Low expression	18 [81.8]	10 [71.4]	28 [77.8]	0.683
		High expression	4 [18.2]	4 [28.6]	8 [22.2]	
RFI		Mean [SD]	11.9 [6.9]	14.8 [7.0]	13.1 [7.0]	0.237
		Post-OP amount steroid groups				
			Group1[ ≤ 600 mg]	Group2[> 600 mg]		
CD8^+^T-cell expression		Low expression	13 [92.9]	15 [68.2]	28 [77.8]	0.115
		High expression	1 [7.1]	7 [31.8]	8 [22.2]	
RFI		Mean [SD]	10.6 [6.2]	14.6 [7.2]	13.1 [7.0]	0.100
		Total-amount steroid groups				
			Group1[ ≤ 800 mg]	Group2[> 800 mg]		
CD8^+^T-cell expression		Low expression	15 [88.2]	13 [68.4]	28 [77.8]	0.236
		High expression	2 [11.8]	6 [31.6]	8 [22.2]	
RFI		Mean [SD]	12.3 [6.9]	13.7 [7.3]	13.1 [7.0]	0.573
		CD8 + T-cell expression				
		Low expression		High expression		
SRC-1 Gene expression		Upregulated	Downregulated	Upregulated	Downregulated	
RFI > 1y	Frequency	1	11	0	6	
Total dose of steroid	Mean	–	1105.29	–	1929.63	
	Std Deviation	–	1028.77	–	1562.46	
	Minimum	–	212.3	–	800	
	Maximum	–	3812.1	–	5028	

*RFI is counted in months. *Fisher exact test

### The relationship between SRC-1 regulation and CD8^+^T-Cells expression with RFI

There was a significantly statistical difference between CD8^+^T-Cell expression and SRC-1 gene regulation in the tumour microenvironment [p-value = 002]. Tumours with high CD8^+^T-cell expression and SRC-1 gene downregulation had late recurrence (Fig. 2f). All tumours with SRC-1 gene upregulation, regardless CD8^+^T-Cell expression, had earlier RFI compared to tumours with downregulated SRC-1 gene. Patients who have received corticosteroids continuously for up to one year showed delayed RFI in tumors with low CD8 + T-Cell expression and downregulated SRC-1 gene (Table 2).

## Discussion

**Fig. 3:**
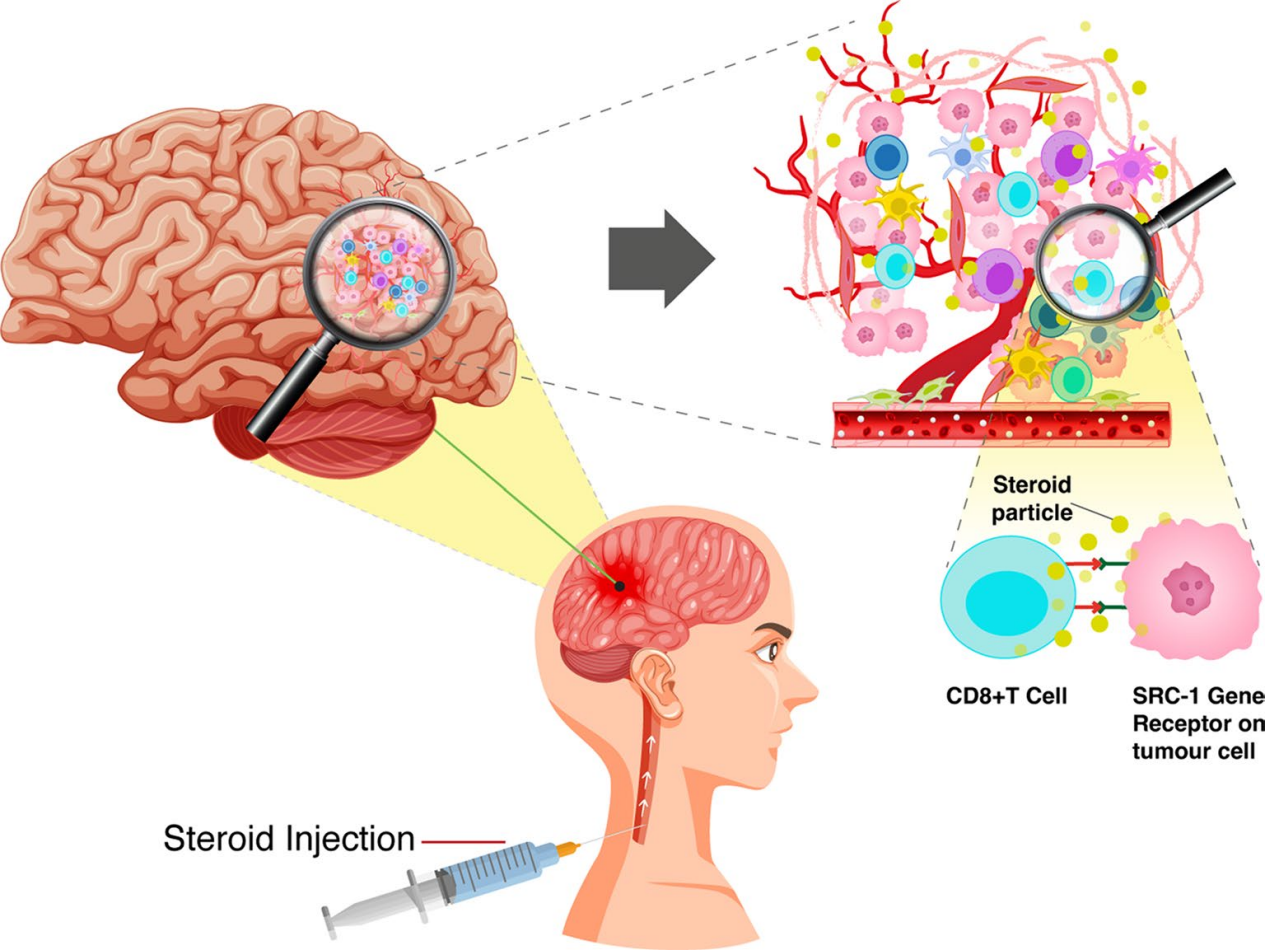
After administering corticosteroid [DEX] to a patient with a brain tumor, the steroid particles reach the tumor microenvironment through BBB. DEX particles either bind to SRC-1 receptors or are diluted in edematous tissue. DEX directly affects SRC-1 gene regulation and indirectly affects cytotoxic T-cells. Tumors with an upregulated SRC-1, had an earlier tumor recurrence compared to those with downregulated SRC-1 gene, regardless of CD8 + T-Cell expression

After administering corticosteroid [DEX] to a patient with a brain tumor, the steroid particles enter the brain tissue through the blood-brain barrier [BBB] and reach the tumor microenvironment. However, the exact timing of how long the steroid particles remain in the tumor microenvironment is unclear, and it is also uncertain what exact reactions the steroid can have, aside from reducing peritumoral edema. The mechanism of how steroid particles interact with SRC-1 receptor and CD8 cytotoxic T-cells in WHO-Grade 4 astrocytoma has never been described. CD8 cytotoxic T-cells may have the ability to suppress tumor cell proliferation and progression; the co-treatment using chemotherapy can diminish CD8 T cells proliferation [25–28, 42, 43]. The only dominant cells that can prevent this mechanism to occur is the tumour associated macrophages [TAMs]. TAMs can mask tumour cells and prevent them being identified by cytotoxic T-cells [29]. Previous studies found that long-term use of corticosteroid after surgery has had a negative impact on patients OS [19, 21]. Our findings did not lead to the same conclusion. We found that the tumor recurrence time did not significantly differ between cases expressing high or low CD8 cytotoxic T-cells, regardless of the amount of injected corticosteroid (Fig. 3). This implies that steroid particles do not have an impact on the functioning of cytotoxic T-cells. It can also be suggested that the steroid molecules may either be diluted in the edematous tissue surrounding the tumor cells or bind with other receptors, thereby preventing them from initiating any auxiliary reaction. One of these gene receptors is SRC-1. SRC-1 expression has been previously detected in astrocytoma [40]. Its expression in tumour microenvironment suggests its possible role in tumour progression. Nevertheless, its association with corticosteroid therapy and cytotoxic T-cells function has never been investigated. When steroid particles reach the tumour microenvironment, they may bind to SRC-1 gene receptor (Fig. 3) Typically, steroid particles are expected to inhibit the regulation of the SRC-1 gene, which would lead to a reduction in its expression in tumor microenvironment. However, in our study, we observed that five tumors showed an upregulated SRC-1 gene, indicating that steroid particles did not bind to the SRC-1 receptors. These tumors, with an upregulated SRC-1 gene, had an earlier tumor recurrence when compared to those with downregulated SRC-1 gene, regardless of CD8 + T-Cell expression. The cases that exhibited late tumor recurrence were only those with downregulated SRC-1 gene (Fig. 2). Among them, 6 cases had high CD8 cytotoxic T-cells, and 11 cases had low CD8 cytotoxic T-cells Table 2. The cases that showed large number of cytotoxic T-cells had an average total dose of a steroid of 1926.63 mg compared to cases with small number of cytotoxic T-cells which had an average total dose of a steroid of 1105.29 mg. This clearly identifies that steroid dose has no effect on T-cell expression in tumour microenvironment and has no significant impact on tumour progression. Corticosteroid therapy directly affects SRC-1 gene regulation and indirectly affect the cytotoxic T-cells. When the SRC-1 gets downregulated or upregulated, the cytotoxic T-cells react aggressively with tumour cells. However, the SRC-1 is considered an independent factor for tumour recurrence. To study SRC-1 gene regulation in glioblastoma patients treated by corticosteroids, in relation with CD8 T cells expression, the use of antisense RNA of SRC-1 gene constitutes one of substantial possibilities. A study conducted by Resnicoff et al. suggests that the regression of established wild-type C6 tumors is feasible through the injection of C6 cells that express an antisense RNA to Insulin growth factor (IGF)-1R RNA. This study implies that there may be practical applications of this approach in SRC-1 regulator gene to test the effectiveness of corticosteroid in glioblastoma [44]. The two primary approaches to regulate expression by recognizing cellular RNAs through antisense mechanisms are single-stranded antisense oligonucleotides and duplex RNAs [45].

Several mechanisms and theories have been proposed in the literature to explain the interaction between corticosteroid therapy [DEX] and tumor progression, but none of them have provided a definitive answer. A microarray analysis on mice glioma samples was performed by Pitter et al., to identify the DEX genes associated mechanism responsible for DEX flaring or resistance. The study showed that DEX has decreased tumour cells proliferation as well as the expression of many cells cycle-related genes [22]. These genes were predicted to be involved in proliferation process, either via cell mitotic mechanism, cycle checkpoints or DNA damage.

The radiotherapy sensitivity can affect the glioma cells proliferation during the cell cycle proliferative stage. Glioma cells are generally more radiosensitive when they are in G2/M stage of the cell cycle and more resistant when they are in G1 stage of the cycle. Any drug injected during this cycle to decrease cellular growth would potentially reduce the radio-sensitivity of all cell population [46]. Glassier et al. found the p21 protein is induced by DEX in glioma cells and slows cell cycle progression [47]. This mechanism is induced or inhibited based on the accumulated dose of DEX that patient receive before or after surgery. In our study, the use of chemotherapy, primarily TMZ, did not show any significant impact on the effect of steroids on patient survival. Gorlia et al.‘s found that patients who received TMZ and radiotherapy were less frequently treated with steroids at the beginning of their treatment. They also concluded that patients who were treated with steroids at the start had a shorter OS [overall survival] [48]. Additionally, it has been demonstrated that DEX acts as an antagonist to TMZ-induced apoptosis in glioblastoma, indicating that the combination of TMZ and DEX may counteract each other in the treatment of glioblastoma [49].

Although the total number of samples analyzed is relatively low, it is important to note that our study is the first to establish a correlation between SRC-1 gene expression and cytotoxic T-cells with RFI in WHO-grade 4 astrocytomas.

## Conclusions

Our findings indicate that: [a] corticosteroid treatment does not affect the function of cytotoxic T-cells infiltration and has no significant impact on tumor progression; [b] tumors with downregulated SRC-1 gene had a later tumor recurrence compared to those with upregulated SRC-1 gene, regardless of cytotoxic T-cell infiltration; [c] corticosteroid treatment directly affects SRC-1 gene regulation and indirectly affects cytotoxic T-cells infiltration.

.

## Data Availability

The datasets generated during and/or analysed during the current study are available from the corresponding author on reasonable request.
